# Growth substrate may influence biofilm susceptibility to antibiotics

**DOI:** 10.1371/journal.pone.0206774

**Published:** 2019-03-14

**Authors:** Dustin L. Williams, Scott R. Smith, Brittany R. Peterson, Gina Allyn, Lousili Cadenas, Richard Tyler Epperson, Ryan E. Looper

**Affiliations:** 1 George E. Wahlen Department of Veterans Affairs, Salt Lake City, UT, United States of America; 2 Department of Orthopaedics, University of Utah, Salt Lake City, UT, United States of America; 3 Department of Pathology, University of Utah, Salt Lake City, UT, United States of America; 4 Department of Bioengineering, University of Utah, Salt Lake City, UT, United States of America; 5 Department of Physical Medicine and Rehabilitation, Uniformed Services University, Bethesda, MD, United States of America; 6 Department of Chemistry, University of Utah, Salt Lake City, UT, United States of America; Thomas Jefferson University, UNITED STATES

## Abstract

The CDC biofilm reactor is a robust culture system with high reproducibility in which biofilms can be grown for a wide variety of analyses. Multiple material types are available as growth substrates, yet data from biofilms grown on biologically relevant materials is scarce, particularly for antibiotic efficacy against differentially supported biofilms. In this study, CDC reactor holders were modified to allow growth of biofilms on collagen, a biologically relevant substrate. Susceptibility to multiple antibiotics was compared between biofilms of varying species grown on collagen versus standard polycarbonate coupons. Data indicated that in 13/18 instances, biofilms on polycarbonate were more susceptible to antibiotics than those on collagen, suggesting that when grown on a complex substrate, biofilms may be more tolerant to antibiotics. These outcomes may influence the translatability of antibiotic susceptibility profiles that have been collected for biofilms on hard plastic materials. Data may also help to advance information on antibiotic susceptibility testing of biofilms grown on biologically relevant materials for future *in vitro* and *in vivo* applications.

## Introduction

**Fig 1:**
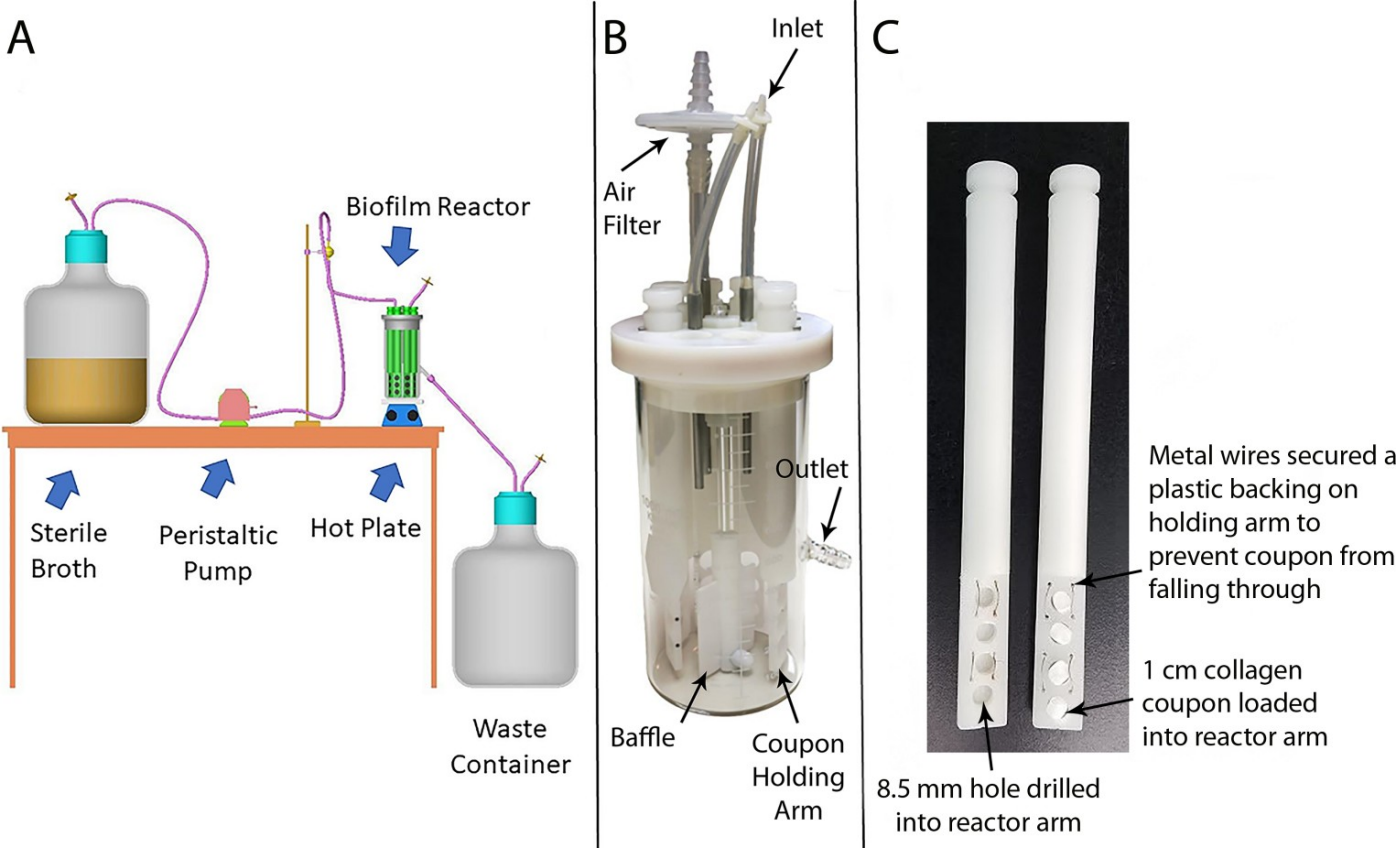
Design of the CDC biofilm reactor and modified holder. (A) Schematic of a general setup of a CDC biofilm reactor for biofilm growth. Source: Biosurface Technologies. (B) Components of a CDC biofilm reactor. (C) Customized rods into which collagen plugs were placed for biofilm growth. Labels detail modifications.

The CDC biofilm reactor has been validated as a robust and repeatable reactor system for elucidating various aspects of biofilm physiology, morphology, growth dynamics and antibiotic susceptibility profiles [1–6]. The reactor was designed with removable coupons that are used to asses biofilm characteristics and properties (Fig 1A). This allows for exposure to shear force and renewable nutrients that optimize biofilm formation by simulating natural environments such as rivers, streams, oral cavities, biomedical device surfaces or industrial systems [7–11]. Coupons are manufactured from a wide variety of materials to mimic such environments. For example, coupons made of iron-based metals or polyvinylchloride (PVC) that simulate the surface of culinary water pipelines are available. Coupons made of polycarbonate, polyetheretherketone, stainless steel, polypropylene, glass or silicone are often used for medical device-related applications [3,4,12–14]. The majority of coupons that have been analyzed consist of relatively smooth, flat surfaces. A modified CDC biofilm reactor was developed in this study such that it held coupons made of highly porous, bioabsorbable collagen (Fig 1B). The rationale for doing so was two-fold.

First, there is currently a paucity of data on antibiotic efficacy against biofilms that are grown on biologically relevant materials. Collagen constitutes 75% of the dry weight of human skin, and is a major component of extracellular matrix and multiple tissue types that have potential to be affected by biofilm formation [15]. It has also been shown that multiple bacterial species adhere to collagen, and may aid in their ability to colonize and potentially infect tissue types [16–19]. The primary question to be answered in this study was whether biofilms that attached to biological material, such as highly porous and complex collagen, displayed similar antibiotic susceptibility profiles to biofilms that grew on relatively smooth, flat surfaces such as polycarbonate. Secondly, the stability of bioabsorbable collagen in the modified reactor was assessed. More specifically, it needed to be confirmed that collagen would not disintegrate/absorb in the timeframe that biofilms were grown on it.

We hypothesized that biofilms of varying species grown on a complex collagen network would be less susceptible to antibiotics as compared to biofilms grown on polycarbonate coupons. The criteria for support of the hypothesis was a difference of 1 log_10_ unit or greater in bacterial counts between coupon types. A secondary aspect of the study was to demonstrate the ability of modified rods to broaden the scope of substrates on which biofilms can be grown in a CDC reactor for future *in vivo* analyses similar to a previously modified reactor [3,14,20–22].

## Materials and methods

### Reagents and materials

Tryptic soy broth (TSB), brain heart infusion (BHI) broth, agar, cation-adjusted Mueller Hinton broth (CAMHB) and phosphate buffered saline (PBS) were purchased from Fisher Scientific (Waltham, MA). Blank polypropylene holders for the CDC reactor were purchased from Biosurface Technologies (Bozeman, MT). Collaform collagen plugs were purchased from Implant Logistics (La Crosse, WI). All antibiotics used were purchased from Sigma Aldrich (St. Louis, MI) or TCI America (Portland, OR). Antibiotics for each bacterium were chosen based on common clinical use. E-Test strips for MIC testing of amoxicillin and erythromycin were purchased from Biomérieux (Durham, NC).

### Bacterial isolates

Bacterial isolates were chosen because of their use in various applications including standards assays and animal models, their known pathogenicity as well as their ability to form well-established biofilms [23–26]. All isolates were purchased from the American Type Culture Collection (ATCC) and included *Staphylococcus aureus* ATCC 6538, *Pseudomonas aeruginosa* ATCC 27853, *Escherichia coli* ATCC 9637, *Acinetobacter baumannii* ATCC BAA 1605, *Bacillus subtilis* ATCC 19659, methicillin-resistant *S*. *aureus* (MRSA) USA 300, MRSA USA 400, *Streptococcus mutans* ATCC 25175, *S*. *epidermidis* ATCC 35984, and carbapenem-resistant *Klebsiella pneumoniae* ATCC BAA-1705. All were subcultured on tryptic soy agar (TSA) or, in the case of some experiments with *S*. *mutans*, BHI agar, and incubated overnight at 37°C. Frozen stocks were maintained in BHI broth with 30% glycerol. Isolates were subcultured and incubated 24–72 hours prior to inoculation in a biofilm reactor.

### Reactor and materials design

Standard polypropylene CDC biofilm reactor rods were used to hold polycarbonate coupons (Fig 1A). Custom rods were made for the collagen plug coupons (Fig 1B and 1C). Four holes of 8.5 mm diameter each were drilled in the lower portion of a blank polypropylene holder (Fig 1C). This diameter was smaller than the diameter of standard reactor coupons (12.7 mm). The size difference was to ensure a tight fit of the collagen in the holders.

Medical grade Collaform collagen was purchased as 1 cm x 2 cm plugs to make the collagen plugs. Collagen plugs were aseptically removed from packaging and cut into coupons with a sterile blade. Coupon size was 1 cm diameter x 0.3 cm height. Coupons were aseptically loaded into modified reactor rods (Fig 1B) that had already been autoclaved. The collagen coupons remained securely in place when exposed to the shear forces in the reactor. Each reactor was assembled inside a sanitized biosafety cabinet.

### Biofilm growth

A 0.5 McFarland standard, which equated to ~7.5 x 10^7^ colony forming units (CFU)/mL, of each bacteria was made from a fresh culture,. One mL of the 0.5 McFarland solution was inoculated into 500 mL of BHI in the CDC biofilm reactor. The reactor was placed on a hot plate set at 34°C and a baffle rotation of 130 rpm for 24 h. After the 24 h batch growth, a continuous flow of 10% BHI was maintained through the reactor at ~6.9 mL/min for an additional 24 h for a total of 48 h of growth.

### Baseline quantification

Two rods were aseptically removed from a reactor and rinsed in 1x PBS. An n = 6 coupons were individually placed in a test tube that contained 2 mL CAMHB, vortexed for 1 min, sonicated for 10 min, then vortexed a final time for approximately 10 sec. A 10-fold dilution series was used to plate bacteria in duplicate on tryptic soy agar (TSA). Agar plates were incubated overnight at 37°C. CFU were counted (the dilution with a range of 20–200 colonies was selected) and used to calculate a baseline of CFU/coupon.

### Antibiotic treatment

The minimum inhibitory concentration (MIC) of each antibiotic was determined against each bacterial strain prior to assessing the antibiotic susceptibility of biofilms. With the exception of amoxicillin and erythromycin against *Streptococcus mutans*, a modified protocol of the Clinical and Laboratory Standards Institute (CLSI) guideline M100 was used. In short, using a fresh overnight culture of bacteria, a 0.5 McFarland standard was made in PBS using a nephelometer. The stock solution was diluted in PBS 1:100 to achieve a concentration of ~7.5 x 10^5^ CFU/mL. A 96-well plate was set up such that a final volume of 100 μL was present in each well. Column 1 served as the negative control of growth (antibiotic only without bacteria added) and Column 11 served as the positive control of growth (bacteria only, no antibiotic).

One hundred μL of CAMHB that contained antibiotic only (64 μg/mL) was pipetted into each well of column 1 to serve as the negative control. Into columns 2–11, 50 μL of CAMHB were added to each well. Subsequently, 50 μL of CAMHB that contained a concentration of 256μg/mL antibiotic were added to each well of column 2 using a multi-channel pipet. The solution was mixed, then 50 μL were removed and added to wells of column 3. This 1:2 dilution process was continued until column 10 and resulted in a range of antibiotic testing from 64 μg/mL to 0.0625 μg/mL. Lastly, into each well of columns 2–11, 50 μL of the bacterial solution was added, with column 11 serving as the positive control. The 96-well plate was covered with adhesive film and incubated 24 h at 37°C. The concentration of antibiotic that inhibited pellet formation or turbidity at the 24 h time point was considered the MIC. The biofilm analysis was performed after the MIC was determined.

An E-Test was used to determine the MIC of amoxicillin and erythromycin against *S*. *mutans*. In short, *S*. *mutans* was cultured on BHI agar and incubated for 48 hours under 5% CO_2_. A 1.0 McFarland standard solution was made in PBS resulting in 2.8 x 10^8^ CFU/mL solution. This was used to make lawns of bacteria on BHI agar by stroking back and forth with an inoculated Q-tip in three directions. E-Test strips for each compound were laid on agar plates after drying (n = 2 per plate, 2 plates per compound). The plates were incubated for 24 hours under 5% CO_2_ and then analyzed per manufacturer’s instructions.

Ann = 5 polycarbonate and n = 6 collagen coupons were aseptically removed from a reactor following a reactor run, rinsed in 1x PBS, and each coupon was individually placed in a test tube that contained 2 mL solution of antibiotic in CAMHB. Each of the antibiotics were tested initially at 50, 100, and 200 μg/mL concentrations. In some instances, data spread needed to be resolved so additional concentrations were tested down to 25 or up to 400, or 600 μg/mL. Coupons were incubated for 24 h after which time each was quantified as described above.

### Scanning electron microscopy imaging

Scanning electron microscopy (SEM) imaging was used to directly observe biofilm morphology and confirm growth on both coupon types. Biofilms were grown following the same protocol as above. Notably, coupons used for SEM imaging to assess morphology were not the same coupons used for the antibiotic testing. This was not possible given the need to fix and process biofilms, which inherently leads to cell death and would have skewed the antibiotics or baseline quantification data.

A rod was aseptically removed from a reactor, rinsed one time in 1x PBS, and each coupon was individually submerged in modified Karnovsky’s fixative (2.5% glutaraldehyde and 4% formaldehyde in 0.2 M PBS, pH 7.4) for ~1 h. The fixed specimens were dehydrated in 100% ethanol for ~1 h, and then air-dried for ~30 min.

Coupons were placed on an SEM stage and secured using double-sided carbon tape, then coated using a Hummer 6.2 gold sputter coater (Anatech LTD). All coupons were imaged with a JEOL JSM-6610 SEM in secondary electron emission mode.

We confirmed that the vortex and sonication process did in fact remove the large majority of biofilm from the surface of the coupons. After a thorough literature review, removal of biofilms from collagen using vortex and sonication did not appear to have been confirmed previously. Thus, biofilms of each bacterium were grown on both materials, samples were rinsed, vortexed, and sonicated as described, and imaged by SEM (n = 3) to determine if the procedure effectively removed biofilm for accurate quantification.

Lastly, n = 3 new and unused (negative control) coupons were soaked in 10% BHI for 48 hrs to compare surface morphologies of the collagen and polycarbonate coupon materials without bacteria on them. Coupons were then aseptically removed and processed as described above for SEM imaging.

### Statistical analysis

Outcome measures (i.e. bacterial counts) were analyzed using an independent sample *t* test for comparisons with alpha level set at 0.05 throughout.

## Results and discussion

Results of experimentation determined whether the hypothesis, that biofilms of varying species grown on a complex collagen network would be less susceptible to antibiotics as compared to biofilms grown on polycarbonate coupons, was supported or unsupported. The criteria for support of the hypothesis was a difference of 1 log_10_ unit or greater in bacterial counts between coupon types.

### Biofilm growth/SEM imaging

**Fig 2:**
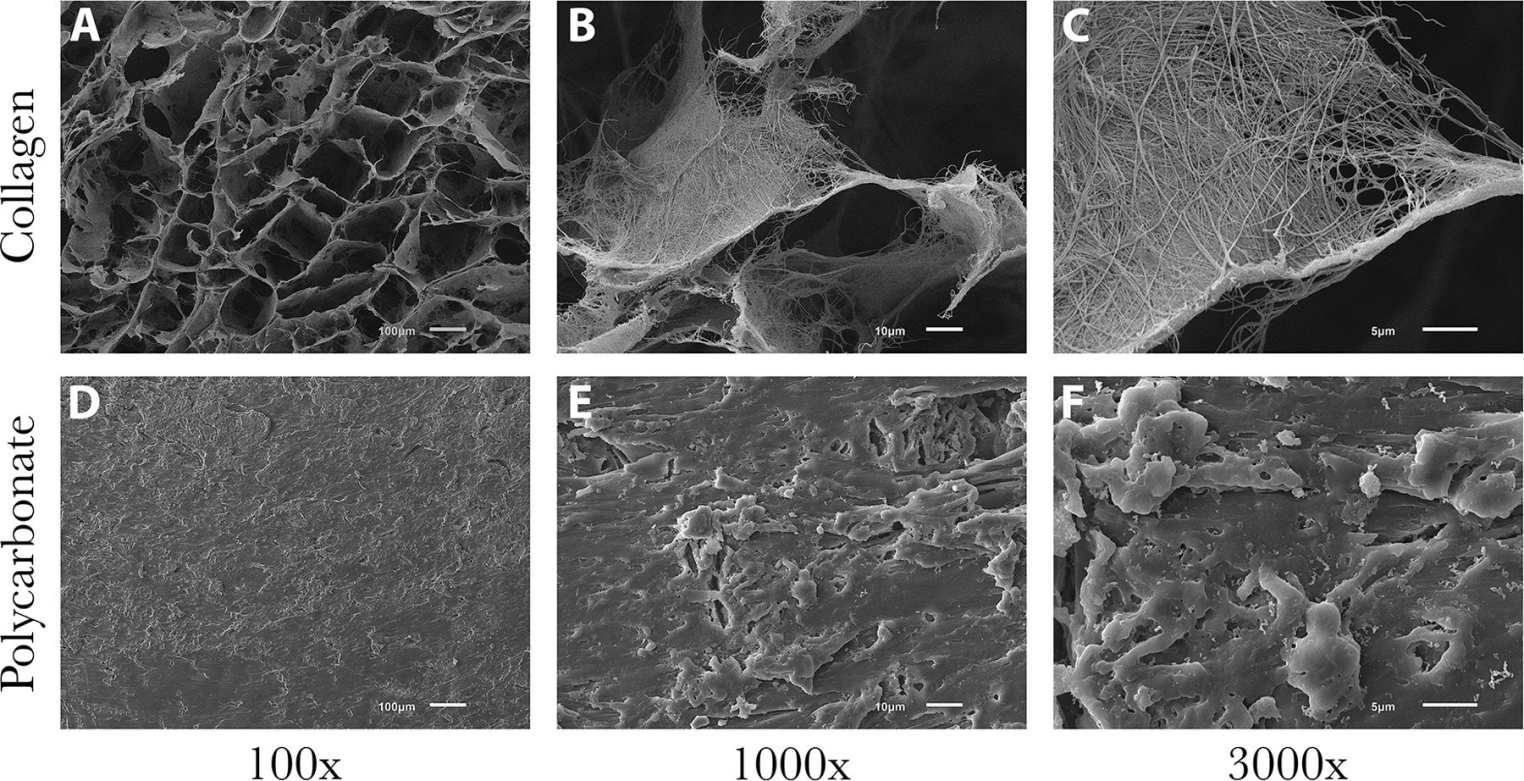
Representative SEM images of collagen and polycarbonate coupon surfaces. (A-C) Surface of a collagen coupon after soaking in broth only (no bacteria present). Deep valleys and ravines were consistent throughout the amorphous structure. (D-F) Surface of a polycarbonate coupon after soaking in broth only (no bacteria present). Ridges and plateaus had an undulating, yet mostly smooth surface, in particular relative to collagen.

**Fig 3:**
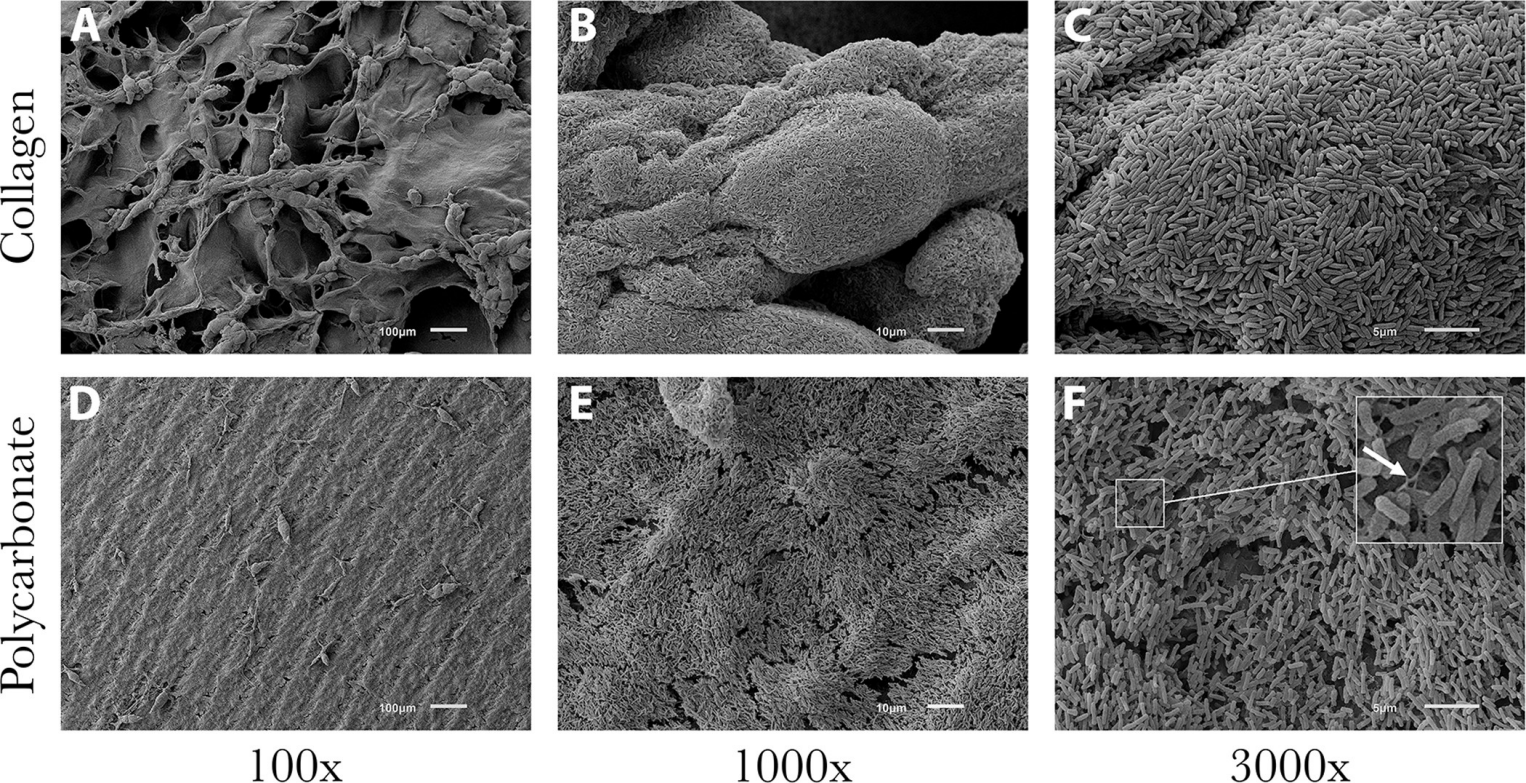
Representative SEM images of *P*. *aeruginosa* ATCC 27853 biofilm formation on both material types. (A-C) Surface of a collagen coupon that had biofilm coverage. Biofilm structure conformed to the polymeric collagen network. As noted in Fig 2, deep valleys and ravines were consistent throughout the amorphous structure. (D-F) Surface of a polycarbonate coupon on which biofilms of *P*. *aeruginosa* ATCC 27853 grew. Biofilm structure was estimated to be greater than 20 cell layers thick. Growth followed the contour of the surface, even displaying the coupon machine marks (100x). Arrow (panel F) indicates extracellular matrix components that were observed.

**Fig 4:**
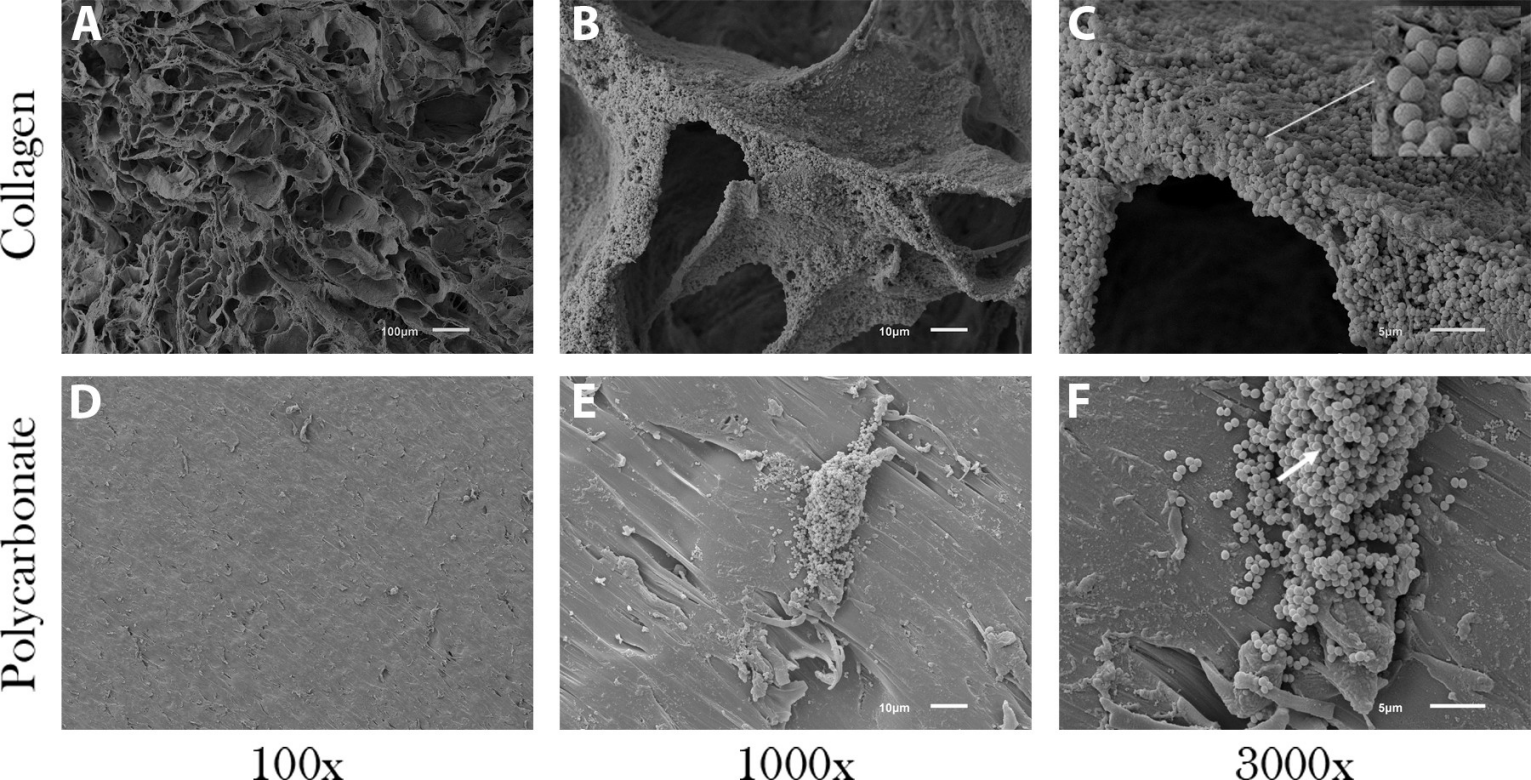
Representative SEM images of MRSA USA 400 biofilm on both material types. (A-C) Surface of collagen with biofilm growth. Biofilm structure resulted in uniform coverage, but did not appear to plume as other staphylococcal isolates did. Rather, MRSA USA 400 displayed sheet-like growth on collagen. (D-F) Surface of a polycarbonate coupon that had sparse coverage, although where biofilm did form, it plumed to an estimated 30 cell layers thick. Arrow (panel F) indicates a biofilm plume.

**Fig 5:**
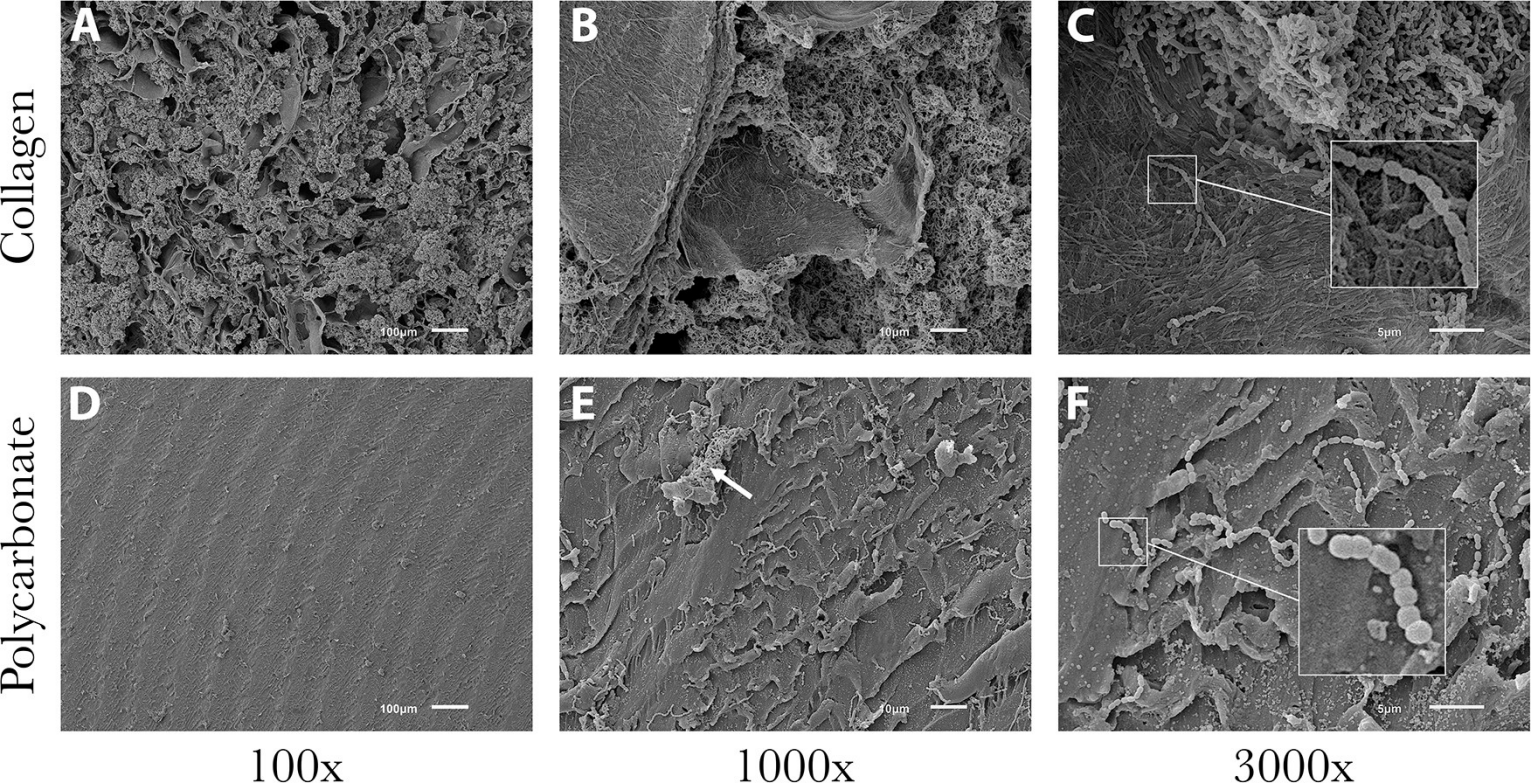
Representative SEM images of *S*. *mutans* ATCC 25175 biofilm formation on both material types. (A-C) Surface of collagen with biofilm coverage. Cells of *S*. *mutans* filled the ravines and crevices of the collagen material. (D-F) In contrast to collagen, *S*. *mutans* grew in small plumes on the surface of polycarbonate with sporadic coverage. Arrow (panel E) indicates a biofilm plume.

SEM images showed that the collagen material alone was amorphous with a polymeric strand network that had deep crevices throughout (Fig 2A–2C). The polycarbonate surface had residual machining marks and isolated regions of raised material with ridges and undulation (Fig 2D–2F). Data showed that biofilms of all species formed on both material types, with some variation in numbers (detailed below). Representative images of *P*. *aeruginosa* ATCC 27853, MRSA USA 400 and *S*. *mutans* ATCC 25175 are provided to show qualitative biofilm formation and morphologies (Figs 3, 4 and 5, respectively).

SEM images showed that *P*. *aeruginosa* ATCC 27853 biofilms were able to form readily on both material types (Fig 3). This isolate did not form into plumes of three-dimensional structure. Rather, communities appeared to grow in stacked sheets (Fig 3). Higher-power images resolved extracellular matrix (ECM)-like fibers extending from one *P*. *aeruginosa* cell to another (Fig 3E and 3F).

Staphylococcal biofilms produced three dimensional structures with vertical stacking morphology wherein cells grew on top of one another in sheet-like structures on collagen that delved deep into the collagen crevices (Fig 4A–4C). On polycarbonate coupons, light plumes of biofilm formed primarily on the stiff, raised regions of the material surface (Fig 4D–4F). On both material types, biofilm growth followed the contour of the material surface (see Fig 4). Extracellular matrix (ECM) development was sporadic, nevertheless fibers of matrix were seen throughout staphylococcal biofilms on both material types (e.g., Fig 4C). Biofilm growth was similar for all staphylococcal isolates (i.e., MRSA USA300, *S*. *aureus* and *S*. *epidermidis)*. *S*. *epidermidis* ATCC 35984 produced the most highly robust biofilms of any isolate tested, appearing extremely thick and canyon-like with visible water channels.

All other species produced biofilms with slight variations in thickness and coverage. In the case of *A*. *baumannii* ATCC BAA 1605, biofilms formed into sheet-like structures on both collagen and polycarbonate coupons (data not shown). Isolated regions with matrix-like materials were observed. Layered sheet-like structures of biofilm were also seen in *E*. *coli* ATCC 6937 and *B*. *subtilis* ATCC 19659 with *B*. *subtilis* having the most significant ECM development on the smooth, flat polycarbonate coupons of all biofilms (data not shown). Carbapenem-resistant *K*. *pneumoniae* ATCC BAA-1705 grew a dense, consistent smooth-layer biofilm with small amounts of ECM on polycarbonate (data not shown). *S*. *mutans* ATCC 25175 grew a scattered monolayer biofilm on polycarbonate and thick plumes of biofilm covering most of the collagen (Fig 5). On both materials there was little ECM.

**Fig 6:**
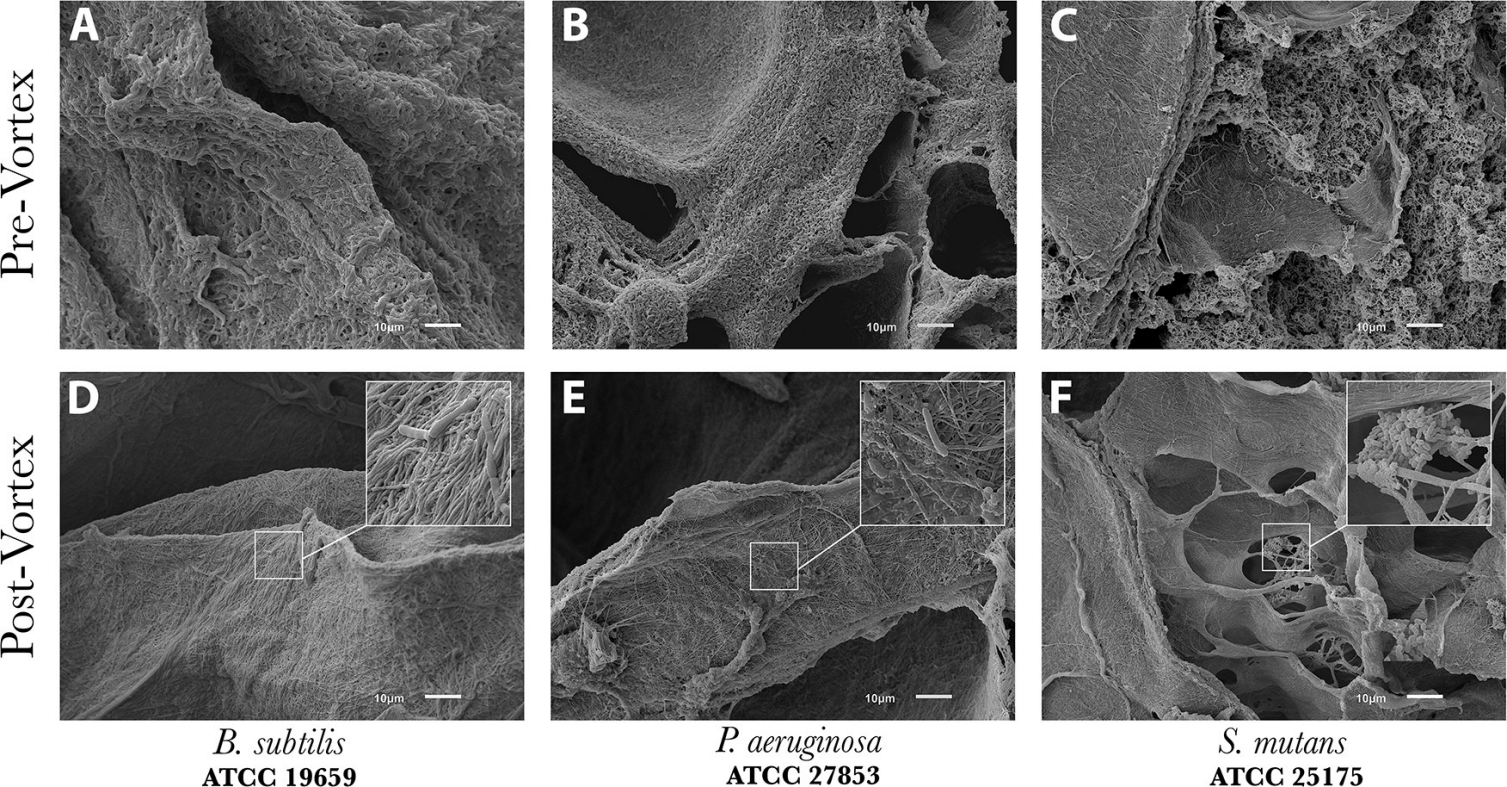
Representative SEM images of collagen coupons with three isolates pre- and post-vortex/sonication to demonstrate the ability of the process to remove bioburden from material surfaces. (A-C) Representative images of biofilm structures prior to vortex and sonication. (D-F) Images of residual cells on collagen after vortex and sonication. Data indicated that biofilms were effectively disrupted/removed with <5% of cells remaining on the surface.

**Fig 7:**
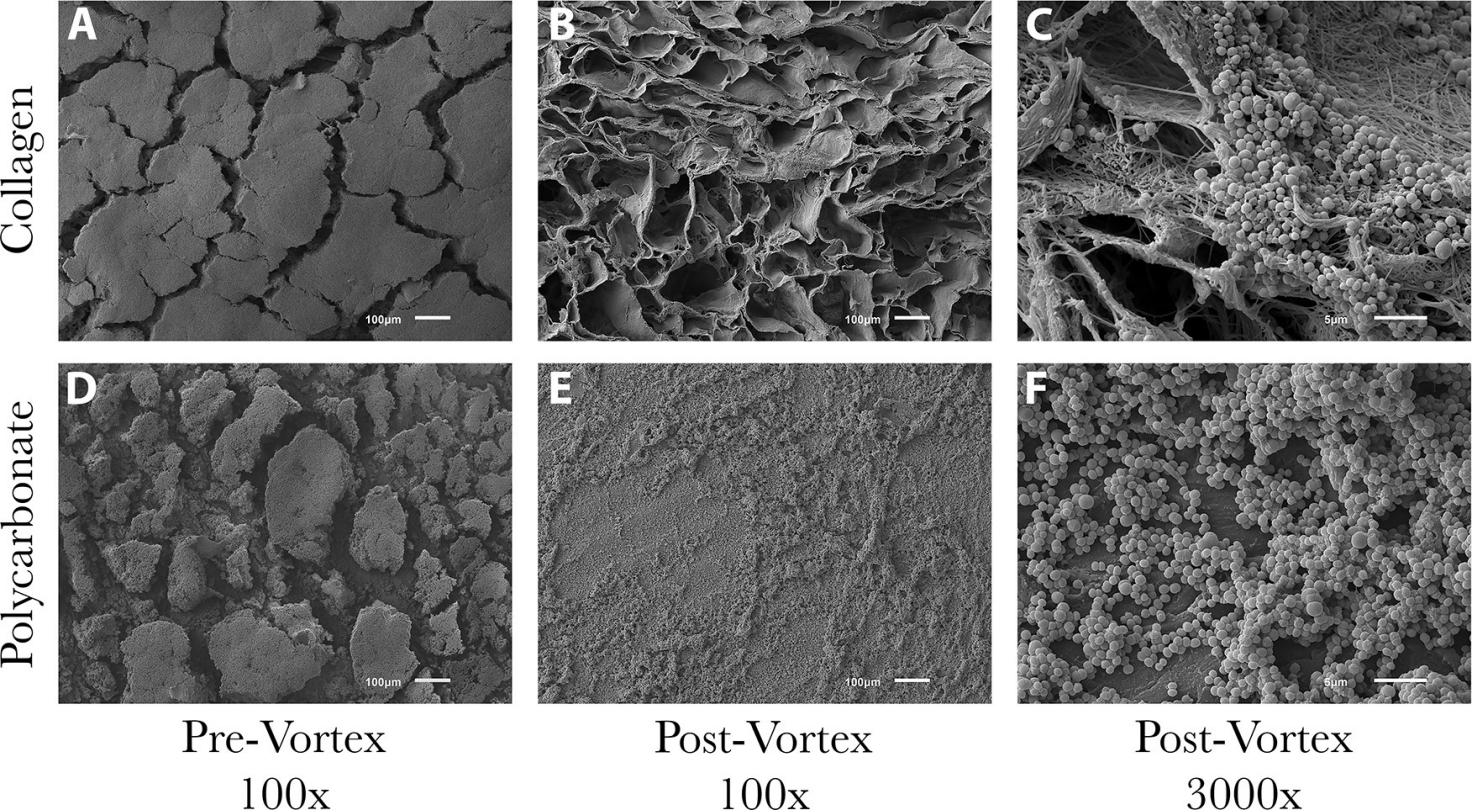
Representative SEM images of *S*. *epidermidis* ATCC 35984 biofilms on collagen and polycarbonate coupons pre- and post-vortex/sonication. (A) Heavy amounts of biofilm formed on collagen coupons, making the surface morphology unobservable. (B-C) Images showed that the large majority of biofilm burden had been removed by vortex/sonication, but clusters of cells still remained. (D) Similar to collagen, large plumes of biofilms formed on polycarbonate coupons with deep ravines. Although these ravines may have formed during the dehydration procedure, it is likely they were sites of water channels that provided fracture points within the biofilm communities. (E-F) Images showed that there was still a fair amount of surface coverage by biofilms post-vortex/sonication. However, it was estimated that there were <5% of cells remaining on the surface similar to other bacterial isolates examined.

SEM images of coupons that had been vortexed and sonicated indicated that the large majority of biofilms had been removed from the surface (Fig 6). This confirmed that when quantifying the CFU/sample, the majority of bacterial cells were accounted for in the 10-fold dilution series. The degree of biofilm removal was similar for all biofilms (Fig 6), with the exception of *S*. *epidermidis* ATCC 35984 (Fig 7). Although there was still a relatively fair amount of *S*. *epidermidis* ATCC 35984 cells that remained on each material type post-vortex/sonication, the levels of biofilm that were present initially were far more than any other isolate (see Figs 3–7). Thus, the reduction was still significant. Based on surface area coverage and reduction of the biofilm layers to a monolayer of cells, it was estimated that less than 5% of cells remained on the surface after vortexing and sonication.

### Baseline quantification and antibiotic treatment

Quantification data of untreated coupons (positive controls of growth) are reported in Table 1. Of the ten isolates examined, six had growth profiles on collagen versus polycarbonate that were statistically significantly different (Table 1). In each case where there was a difference, more growth was present on collagen compared to polycarbonate (Table 1).

**Table 1:** Baseline (control) quantifications of coupons.

Isolate	Average Log_10_ Transformed CFU/Polycarbonate Coupon (n = 6)	Average Log_10_ Transformed CFU/Collagen Coupon (n = 6)	Statistical Significance (p value)
*S*. *aureus* ATCC 6538	9.37 ± 0.07	9.36 ± 0.28	p = 0.89
*P*. *aeruginosa* ATCC 27853	8.02 ± 1.01	8.16 ± 0.15	p = 0.85
*E*. *coli* ATCC 9637	7.40 ± 0.49	8.95 ± 0.76	p = 0.002
*A*. *baumannii* ATCC BAA 1605	8.42 ± 0.36	7.70 ± 0.36	p = 0.006
*B*. *subtilis* ATCC 19659	6.82 ± 0.70	7.28 ± 0.20	p = 0.15
MRSA USA 300	7.49 ± 0.42	9.61 ± 0.34	p = 0.001
MRSA USA 400	8.19 ± 0.45	9.32 ± 0.19	p = 0.001
*S*. *mutans* ATCC 25175	7.73 ± 0.93	8.70 ± 0.36	p = 0.05
*S*. *epidermidis* ATCC 35984	9.15 ± 0.67	9.16 ± 0.33	p = 0.96
*K*. *pneumoniae* ATCC BAA 1705	7.14 ± 0.24	8.61 ± 0.23	p = 0.001

MICs for each antibiotic against the bacterial species are reported in Table 2. Only one isolate (*K*. *pneumoniae*) was resistant to the antibiotics tested. Despite lack of susceptibility against *K*. *pneumoniae*, data are still provided to support the test method, i.e., to show that if an isolate was not susceptible, no biofilm reduction would have be present, but when susceptible, reduction in biofilm numbers could be considered accurate. Biofilm susceptibility outcomes for both material types are summarized below. Full results are provided in Table 3.

**Table 2:** Bacteria and the antibiotics they were tested against, including MIC for each.

Bacteria	Antibiotic	MIC (μg/mL)
*Staphylococcus aureus* ATCC 6538	Ciprofloxacin	0.25
	Cefazolin	≤0.0625
*Pseudomonas aeruginosa* ATCC 27853	Tobramycin	0.25
	Ciprofloxacin	0.5
*Escherichia coli* ATCC 9637	Ceftriaxone	0.0625
	Ciprofloxacin	≤0.0625
*Acinetobacter baumannii* ATCC BAA 1605	Imipenem	16
	Colistin	1
*Bacillus subtilis* ATCC 19659	Ciprofloxacin	0.5
	Vancomycin	0.25
Methicillin-resistant *Staphylococcus aureus* (MRSA) USA 300	Vancomycin	2
	Daptomycin	8
Methicillin-resistant *Staphylococcus aureus* (MRSA) USA 400	Vancomycin	1
	Daptomycin	8
*Streptococcus mutans* ATCC 25175	Erythromycin	0.047
	Amoxicillin	0.064
*Staphylococcus epidermidis* ATCC 35984	Vancomycin	1
	Ciprofloxacin	0.5
Carbapenem-resistant *Klebsiella pneumoniae* ATCC BAA-1705	Ceftriaxone	>250
	Imipenem/Cilastatin	32

**Table 3:** Average of Log_10_ transformed CFU/sample for each species and material at each concentration of antibiotic tested. Unless otherwise noted (at times coupons fell out of the rod in the reactor and were not included in analysis), n = 6 collagen coupons were analyzed and n = 5 polycarbonate coupons were analyzed. It was hypothesized that biofilms of varying species grown on a complex collagen network would be less susceptible to antibiotics as compared to biofilms grown on polycarbonate coupons. The criteria for support of the hypothesis was a difference of 1 log_10_ unit or greater in bacterial counts between coupon types.

Bacteria	Antibiotic	Material	25 μg/mL	50 μg/mL	100 μg/mL	200 μg/mL	400 μg/mL	600 μg/mL	Hypothesis supported? (Y/N)
***S*. *aureus* ATCC 6538**	Ciprofloxacin	Collagen	-	8.89±0.15	8.62±0.07	8.50±0.13	8.35±0.16	-	No
		Polycarbonate	-	8.46±0.45	8.50±0.21	7.47±0.29	8.31±0.15	-	
	Cephazolin	Collagen	-	-	9.34±0.27	8.90±0.21	9.02±0.12	-	No
		Polycarbonate	-	-	9.13±0.83	8.90±0.17	8.72±0.08	-	
***P*. *aeruginosa* ATCC 27853**	Tobramycin	Collagen	5.26±0.89	*3.99±0.33	§0±0	-	-	-	Yes
		Polycarbonate	4.88±0.33	1.72±1.80	*0±0	-	-	-	
	Ciprofloxacin	Collagen	-	4.29±0.76	4.64±0.90	4.12±0.49	-	-	Yes
		Polycarbonate	-	4.11±0.34	3.87±0.74	1.77±1.62	-	-	
***E*. *coli*** **ATCC 9637**	Ceftriaxone	Collagen	-	§6.32±1.21	§3.60±3.65	4.17±3.53	-	-	Yes
		Polycarbonate	-	0±0	*0±0	0±0	-	-	
	Ciprofloxacin	Collagen	7.00±0.21	6.30±0.43	3.52±2.87	-	-	-	Yes
		Polycarbonate	5.14±0.10	1.01±2.27	0±0	-	-	-	
***A*. *baumannii* ATCC BAA 1605**	Imipenem	Collagen	-	8.38±0.12	7.31±0.40	7.31±0.38	-	-	No
		Polycarbonate	-	8.42±0.05	7.54±0.16	7.20±0.24	-	-	
	Colistin	Collagen	-	4.91±2.57	0±0	0±0	-	-	No
		Polycarbonate	-	4.28±2.61	0±0	0±0	-	-	
***B*. *subtilis* ATCC 19659**	Ciprofloxacin	Collagen	-	5.71±0.64	3.99±0.35	0±0	-	-	Yes
		Polycarbonate	-	5.58±0.76	0±0	0±0	-	-	
	Vancomycin	Collagen	-	5.68±0.46	4.64±0.36	5.13±0.41	-	-	Yes
		Polycarbonate	-	5.57±0.31	0±0	1.84±2.52	-	-	
**MRSA** **USA 300**	Vancomycin	Collagen	-	-	8.74±0.49	9.42±0.46	9.27±0.07	-	Yes
		Polycarbonate	-	-	6.61±0.48	6.52±0.68	2.42±2.24	-	
	Daptomycin	Collagen	-	7.41±0.70	7.12±0.35	1.30±1.43	-	-	Yes
		Polycarbonate	-	2.22±2.14	1.93±1.79	0±0	-	-	
**MRSA** **USA 400**	Daptomycin	Collagen	-	7.48±0.25	6.68±0.53	5.50±0.83	-	-	Yes
		Polycarbonate	-	4.38±1.03	*3.27±1.03	1.62±2.36	-	-	
	Vancomycin	Collagen	-	8.87±0.37	9.00±0.19	8.78±0.50	-	-	Yes
		Polycarbonate	-	6.54±0.42	6.48±0.56	7.17±1.05	-	-	
***S*. *mutans* ATCC 25175**	Erythromycin	Collagen	-	8.89±0.50	6.80±0.85	5.97±0.88	-	-	Yes
		Polycarbonate	-	1.23±2.75	0±0	0±0	-	-	
	Amoxicillin	Collagen	-	8.05±0.20	8.13±0.39	7.77±0.23	-	-	Yes
		Polycarbonate	-	4.98±0.33	4.81±0.38	5.23±0.28	-	-	
***S*. *epidermidis* ATCC 35984**	Vancomycin	Collagen	-	8.33±0.14	§8.45±0.16	8.47±0.09	-	-	No
		Polycarbonate	-	8.66±0.35	8.62±0.10	8.49±0.15	-	-	
	Ciprofloxacin	Collagen	-	8.32±0.17	7.46±0.06	6.34±0.05	-	-	No
		Polycarbonate	-	8.55±0.10	6.76±0.34	6.07±0.11	-	-	
***K*. *pneumoniae* ATCC BAA-1705**	Ceftriaxone	Collagen	-	9.58±0.07	8.84±0.51	9.23±0.14	8.99±0.15	8.74±0.34	N/A
		Polycarbonate	-	9.19±0.15	8.90±0.42	8.91±0.27	8.37±0.18	*8.08±0.16	
	Imipenem/ Cilastatin	Collagen	-	9.29±0.07	9.63±0.57	9.23±0.18	9.10±0.09	8.97±0.10	N/A
		Polycarbonate	-	8.74±0.15	9.41±0.25	8.84±0.23	8.82±0.16	8.75±0.21	

* n = 4 coupons tested

§ n = 5 coupons tested

Biofilms of *S*. *aureus* ATCC 6538 grown on collagen or polycarbonate were minimally affected by ciprofloxacin, but did show a statistically significant difference with biofilms on collagen having lower reduction than those on polycarbonate at 200 μg/mL (p = 0.001; see Table 3). Susceptibilities were the same when exposed to cefazolin across a range of concentrations (Table 3; p>0.3 in both cases). Although there was a statistically significant difference in one case with ciprofloxacin, the difference was minimal and was not 1 log_10_ unit or greater. This did not meet the criteria for supporting the hypothesis. The hypothesis likewise was not supported with cefazolin.

In the case of *P*. *aeruginosa* ATCC 27853, there was an approximately 4–5 Log_10_ reduction following exposure to ciprofloxacin at 50, 100, and 200 μg/mL (Table 3). Susceptibility of biofilms to ciprofloxacin on collagen was significantly less compared to those on polycarbonate (e.g., p = 0.03 at 200 μg/mL). Biofilm susceptibility to tobramycin had similar outcomes. Tobramycin resulted in a 4–5 Log_10_ reduction at 50 μg/mL and complete kill at 100 μg/mL for both coupon types, with susceptibility being greater on polycarbonate coupons compared to collagen (Table 3; e.g., p = 0.046 at 50 μg/mL). The hypothesis was supported with both antibiotics in this data set.

Biofilms of *E*. *coli* ATCC 9637 had the most notable differences in susceptibility profiles between collagen and polycarbonate growth (Table 3). In all data sets, both ciprofloxacin and ceftriaxone were more effective against biofilms on polycarbonate than those on collagen with ceftriaxone having more polarized effect than ciprofloxacin (Table 3). Differences between collagen and polycarbonate testing were all statistically significantly different. As a representative example, p = 0.001 for ciprofloxacin at 100 μg/mL. The hypothesis was most strongly supported in this data set, in particular with ceftriaxone.

Biofilms of *A*. *baumannii* ATCC BAA 1605 were highly susceptible to colistin, with an 8 Log_10_ reduction (undetectable growth) on both collagen and polycarbonate coupons at 100 μg/mL, and near complete kill at 200 μg/mL (Table 3). The difference in CFU/coupon was significantly different from baseline controls (p<0.005 in all cases), but there was no statistically significant difference in the number of CFU/coupon between coupon types treated with colistin at 50 μg/mL (p = 0.371). These results indicated that efficacy of colistin against *A*. *baumannii* biofilms was similar on both material types. Imipenem had minimal effect on biofilms on either material type up to 200 μg/mL, and indicated that biofilm susceptibility was similar on both materials (p = 0.590). The hypothesis was not supported in any case for *A*. *baumannii* and the antibiotics tested.

Biofilms of *B*. *subtilis* ATCC 19659 were found to be more susceptible to vancomycin on polycarbonate than collagen (Table 3; e.g., p = 0.001 at 100 μg/mL). Although there was no detectable growth of biofilms on polycarbonate exposed to vancomycin at 100 μg/mL, an anomaly was observed on polycarbonate coupons treated with vancomycin at 200 μg/mL; two of five coupons had growth. The experiment was repeated with similar outcomes, resulting in a large standard deviation at that concentration (Table 3). Biofilms on polycarbonate treated with ciprofloxacin were reduced by >6.5 Log_10_ units (undetectable growth) at 100 μg/mL, whereas those on collagen were reduced by only ~3 Log_10_ units (p = 0.001). At 200 μg/mL, ciprofloxacin eradicated biofilms completely on both polycarbonate and collagen. At 50 μg/mL, vancomycin and ciprofloxacin each reduced biofilms by ~1 Log_10_ unit on polycarbonate and collagen coupons (p>0.1 in all cases; Table 3). At concentrations above 50 μg/mL, the hypothesis was supported for both antibiotics tested in this data set.

In the case of MRSA USA 300, daptomycin was more effective at eradicating biofilms on polycarbonate compared to collagen (p<0.001 in all cases), in particular at 400 μg/mL (Table 3). Vancomycin was nearly ineffective against biofilms on collagen (see Table 3) and reduced biofilms on polycarbonate coupons to a much greater degree (e.g., p = 0.002 at 400 μg/mL).

Similar to USA300 data, daptomycin was more effective at eradicating MRSA USA 400 biofilms on polycarbonate compared to collagen (e.g., p = 0.003 at 100 μg/mL). Biofilms on collagen showed no significant reduction by vancomycin at any concentration (Table 3) and on polycarbonate showed a reduction of approximately 1.5 Log_10_ units by vancomycin at 100 μg/mL, which was significant (p = 0.001). Outcomes indicated that vancomycin was more effective at eradicating MRSA USA 400 biofilms on polycarbonate compared to collagen.

Biofilms of *S*. *mutans* ATCC 25175 on collagen showed no significant reduction from baseline growth and no difference when exposed to 50 μg/mL erythromycin (p = 0.471). Increasing concentrations of erythromycin reduced biofilms on collagen by approximately 2–3 log_10_ units (Table 3). On polycarbonate coupons, erythromycin reduced biofilms by approximately 6 log_10_ units at 50 μg/mL (see Tables 1 and 3), and further reduced biofilms to below detectable levels at 100 and 200 μg/mL (Table 3) indicating that erythromycin was more effective against biofilms on polycarbonate than collagen. Biofilms on collagen were minimally affected by amoxicillin (Table 3.) However, biofilms on polycarbonate showed significant reductions of approximately 2.5 Log_10_ units by amoxicillin at all concentrations (e.g., p = 0.001 at 200 μg/mL). These data indicated that amoxicillin was more effective at eradicating *S*. *mutans* biofilm on polycarbonate compared to collagen, which contrasted the results of erythromycin. Thus, outcomes with amoxicillin and erythromycin supported the hypothesis.

Biofilms of *S*. *epidermidis* ATCC 35984 showed minimal reduction against biofilms on collagen or polycarbonate when treated with vancomycin, indicating that vancomycin was equally ineffective at eradicating *S*. *epidermidis* biofilms on both material types. Biofilms on collagen showed a reduction of approximately 1.5 Log_10_ units by ciprofloxacin at 100 μg/mL and approximately 2.5 Log_10_ units at 200 μg/mL (Table 3). Biofilms on polycarbonate were reduced by approximately 2.5 Log_10_ units with ciprofloxacin at 100 μg/mL and approximately 3 Log_10_ units at 200 μg/mL. There was a statistical difference between collagen and polycarbonate outcomes with ciprofloxacin (e.g., p = 0.004 at 200 μg/mL). However, despite statistical significance, the difference was less than 1 log_10_ unit, and did not meet the criteria for supporting the hypothesis. Thus, the hypothesis was not supported with ciprofloxacin nor vancomycin.

In the case of *K*. *pneumoniae* ATCC BAA-1705, ceftriaxone and imipenem/cilastatin had minimal effect against biofilms on collagen or polycarbonate. There was about 1 Log_10_ more bacteria that grew on collagen compared to polycarbonate. In all cases, there were more bacteria on coupons in treatment groups than there were on positive controls. These data neither supported nor unsupported the hypothesis as the isolate was resistant to both antibiotics, yet were included to provide a comparison of outcomes when susceptibility was not present.

Taken together, data indicated that in 12/18 cases the hypothesis was supported. *K*. *pneumoniae* data were not included in the outcome as biofilms treated with antibiotics had more growth than positive controls.

Biofilms can be grown on a wide variety of surfaces, materials and exposed to myriad environmental conditions. One of the most common reactor systems to grow biofilms is the CDC biofilm reactor, which is beneficial in that it is a robust system that can be modified to hold a broad variety of coupon types [1,3,5]. Its utility spans a broad scope of research and development. The antibiotic susceptibility of multiple bacterial species was assessed in this study with specific focus on determining whether biofilm growth on coupons made of a complex collagen material or on a relatively smooth, non-complex polycarbonate surface would influence susceptibility. There is significant literature on susceptibility profiles of biofilms grown on hard, relatively smooth polymer surfaces, but a paucity of data for susceptibility on more complex, biologically-relevant materials such as collagen. This disparity provided the rationale for analysis. Understanding susceptibility profiles is important given that clinical antibiotics used in human applications may find utility in targeting biofilms that have significant and direct contact with soft and hard tissue, a primary constituent of which is collagen [27–29].

One component of the study was to determine whether bioabsorbable collagen would dissolve/disintegrate in the modified CDC biofilm reactor. Results indicated that the collagen did not dissolve/disintegrate within the time frame of growth. Indeed, additional data further suggested that collagen was stable to culture conditions for as long as 8 days in the reactor. This demonstrated that growth on bioabsorbable collagen could be performed in the CDC reactor and allows for future applications of this collagen biofilm system toward *in vitro* and *in vivo* applications. For example, in a diabetic pig wound study, biofilms are currently being grown on collagen coupons for inoculation and analysis.

Biofilm susceptibility data indicated that in a slight majority of instances (12/18), the general hypothesis—that biofilms grown on polycarbonate would be more susceptible to antibiotics than those grown on collagen—was supported. Pairings that supported the hypothesis were tobramycin and ciprofloxacin against *P*. *aeruginosa* ATCC 27853, ceftriaxone and ciprofloxacin against *E*. *coli* ATCC 9637, vancomycin and ciprofloxacin against *B*. *subtilis* ATCC 19659, vancomycin and daptomycin against the methicillin-resistant *S*. *aureus* strains USA 300 and USA 400, and erythromycin and amoxicillin against *S*. *mutans* ATCC 25175.

Pairings that did not support the hypothesis were ciprofloxacin and cefazolin against *S*. *aureus* ATCC 6538, colistin and imipenem against *A*. *baumannii* ATCC BAA 1605, erythromycin against *S*. *mutans* ATCC 25175, and ciprofloxacin and vancomycin against *S*. *epidermidis* ATCC 35984. Carbapenem-resistant *K*. *pneumoniae* ATCC BAA 1705 was resistant to both ceftriaxone and imipenem/cilastatin. Factors that may have influenced the lack of hypothesis support include biofilm morphology. Although SEM images provided surface morphology information, no experiments were performed to determine the state of a biofilm core and its resilience to antibiotics. The depth to which biofilms developed in collagen was also not determined. Persister cells deep in the core of the biofilms within the complex collagen substrate may have contributed to the varying susceptibilities. Biofilms were all grown under similar conditions and for the same amount of time (48 hr) which was consistent with published standards, yet maturation may vary by species. Biofilms that develop over a longer period of time may more closely replicate biofilms in clinical environments and thus potentially influence susceptibility profiles [30]. Broth type and time to biofilm maturation could be factors that affected susceptibility on different substrates. Controlling for biomass on each substrate to confirm that biofilms receive similar dosages would also be important to consider for future work. Time and concentration of antibiotic exposure can also be analyzed in future work to determine what influence these have on more significant eradication of biofilms.

Baseline quantification data indicated that five of the ten isolates had similar amounts of biofilm. The five that did not were, *E*. *coli*, MRSA USA300, MRSA USA400, *S*. *mutans*, and *K*. *pneumoniae*, which formed biofilms more readily and to a greater degree on collagen coupons compared to polycarbonate. In four of those cases (*E*. *coli*, MRSA 300, MRSA 400, and *S*. *mutans*), reduction of biofilms on polycarbonate coupons was significantly greater than on collagen coupons by one or both antibiotics. These data highlighted the importance of confirming efficacy profiles of antibiotics against biofilms on material types that may be relevant to a given application. As in the majority of instances biofilms were more tolerant to antibiotics on collagen than on hard surface polycarbonate, for applications of clinical relevance, it may be beneficial to assess antibiotic biofilm efficacy profiles against an isolate(s) in the presence of biologically relevant materials.

The question was posed whether baseline numbers were affected by the rinse process. More specifically, if biofilms adhered to collagen with greater mechanical strength compared to polycarbonate, the rinse process may have removed more biofilm from polycarbonate prior to quantification and thus skewed the outcomes. To determine if a difference existed, data were collected with additional CDC reactor runs as per the protocol outlined in the Methods. *S*. *aureus* ATCC 6538 and *P*. *aeruginosa* ATCC 27853 were grown on collagen and polycarbonate coupons as representative isolates. After the rinse step, 100 μL aliquots of each solution was quantified using a 10-fold dilution series. Data showed that the number of bacteria in collagen and polycarbonate rinse solutions were the same, suggesting that data were not skewed by the rinse process. A second aspect was considered; detergent such as Tween 80 is often used to improve biofilm removal from surfaces. Tween 80 was not used as part of the quantification process in this study. *S*. *aureus* ATCC 27853 and *P*. *aeruginosa* again served as representative isolates and were grown on collagen or polycarbonate coupons. Coupons were quantified in CAMHB with 0.1% Tween 80, or CAMHB alone. Data indicated that bacteria quantified in CAMHB alone had approximately 0.5 log_10_ units more CFU/coupon compared to those quantified in CAMHB with 0.1% Tween 80. Under the quantification conditions used, the use of Tween 80 didn’t appear to affect quantification.

Higher concentrations of antibiotic were not tested given that the primary outcome measure was to determine if susceptibility was similar on two material types, not to determine the concentration of antibiotic that would reduce biofilms to a specific level. Although the reduction of biofilms was not a primary outcome measure, if data were to be applied to clinically relevant paradigms, this would be an important and application-dependent factor.

## Conclusion

In conclusion, susceptibility of biofilms to antibiotics was significantly different in the majority of instances following 48 h of growth on biologically relevant collagen or polycarbonate material. Although there are only speculative reasons why some antibiotic-bacterial pairing produce significant differences in biofilm susceptibility on different substrates, this study demonstrates that there can be differences in susceptibility. Moving forward, these data are valuable for advancing information on antibiotic susceptibility testing of biofilms grown on a variety of material types and encourage the independent evaluation of susceptibilities on study-relevant materials.

## Supporting information

S1 Table Raw microbiological data for *S*. *aureus* ATCC 6538. (XLSX)

S2 Table Raw microbiological data for *P*. *aeruginosa* ATCC 27853. CFU numbers with decimal points are due to quadruplicate platings that were averaged.
(XLSX)

S3 Table Raw microbiological data for *E*. *coli* ATCC 9637. (XLSX)

S4 Table Raw microbiological data for *A*. *baumannii* ATCC BAA 1605. (XLSX)

S5 Table Raw microbiological data for *B*. *subtilis* ATCC 19659. (XLSX)

S6 Table Raw microbiological data for MRSA USA300. (XLSX)

S7 Table Raw microbiological data for MRSA USA400. (XLSX)

S8 Table Raw microbiological data for *S*. *mutans* ATCC 25175. (XLSX)

S9 Table Raw microbiological data for *S*. *epidermidis* ATCC 35984. (XLSX)

S10 Table Raw microbiological data for *K*. *pneumoniae* ATCC BAA-1705. (XLSX)
